# The Brazilian Portuguese version of the Work Productivity and Activity Impairment – General Health (WPAI-GH) Questionnaire

**DOI:** 10.1590/S1516-31802006000600005

**Published:** 2006-11-01

**Authors:** Rozana Mesquita Ciconelli, Patrícia Coelho de Soárez, Clarissa Campos Guaragna Kowalski, Marcos Bosi Ferraz

**Keywords:** Questionnaires, Reproducibility of results, Cross-cultural comparison, Efficiency, Quality of life, Questionários, Reprodutibilidade dos testes, Comparação transcultural, Eficiência, Qualidade de vida

## Abstract

**CONTEXT AND OBJECTIVE:**

It is still difficult to measure work productivity losses caused by health problems. Despite the importance given to this issue over the last few years, most instruments for performing this task are available only in the English language. This study translated the Work Productivity and Activity Impairment - General Health (WPAI-GH) Questionnaire into Brazilian Portuguese, adapted it cross-culturally and evaluated its reliability and validity.

**DESIGN AND SETTING:**

Cross-sectional survey to test scale reliability and validity, at São Paulo Hospital and the clinic of the Rheumatology division of Universidade Federal de São Paulo — Escola Paulista de Medicina (Unifesp-EPM).

**METHODS:**

Data were obtained from a survey that incorporated the WPAI-GH, short form-36 (SF-36) and some demographic questions. The questionnaires were administered by interview to 100 subjects.

**RESULTS:**

Descriptive statistics was used to characterize the subjects. The intraclass correlation coefficient and Cronbach's alpha were used to assess the reliability and internal consistency of the instrument. Intraclass correlation coefficients from 0.79 to 0.90 indicated good reliability. Cronbach's alpha of 0.74 indicated good internal consistency. Pearson's correlation coefficient was used to assess validity. There were significant positive relationships between the WPAI-GH and SF-36.

**CONCLUSION:**

The Brazilian Portuguese version of the WPAI-GH is a reliable and valid measurement tool and may be useful for those who seek to measure the impact on productivity of health problems among populations of Brazilian employees.

## RESUMO

**CONTEXTO E OBJETIVO:**

Mensurar a perda de produtividade devida a problemas de saúde ainda é uma tarefa difícil. Apesar da importância dada ao tema nos últimos anos, a maioria dos instrumentos disponível para realizar tal tarefa está disponível somente na língua inglesa. Este estudo traduziu para o português do Brasil, fez a adaptação cultural e avaliou a confiabilidade e validade do questionário Work Productivity and Activity Impairment-General Health (WPAI-GH).

**TIPO DE ESTUDO E LOCAL:**

Avaliação transversal da validade e confiabilidade dos domínios, realizada no Hospital São Paulo e na clínica da Disciplina de Reumatologia da Universidade Federal de São Paulo — Escola Paulista de Medicina (Unifesp-EPM).

**MÉTODOS:**

Dados foram obtidos através de um levantamento que incluiu o WPAI-GH, SF-36 e algumas questões demográficas. Os questionários foram administrados na forma de entrevista com 100 sujeitos.

**RESULTADOS:**

Estatística descritiva foi usada para caracterizar os sujeitos. Para avaliar a confiabilidade e a consistência interna do instrumento, foram usados os coeficientes de correlação intraclasse e o alfa de Cronbach. O coeficiente de correlação intraclasse de 0,79 a 0,90 indicou uma boa confiabilidade. O alfa de Cronbach de 0.74 indicou uma boa consistência interna. O coeficiente de correlação de Pearson foi usado para avaliar a validade. Houve correlação positiva e significativa entre o WPAI-GH e o SF-36.

**CONCLUSÃO:**

A versão do WPAI-GH para o português do Brasil é um instrumento que possui validade e reprodutibilidade e pode ser útil para aqueles que procuram medir o impacto dos problemas de saúde na produtividade de uma população de trabalhadores brasileiros.

## INTRODUCTION

It has been amply demonstrated how individuals’ health conditions affect their productivity.^1-3^ Problems with employees’ health generally imply decreases in their activities and reduced productivity at work.

To measure the impact of diseases on productivity, several health-related workplace productivity loss instruments have been developed.^4-6^ Most of the time, these instruments relate to individuals who are working on a formal basis or receiving regular pay and they do not include voluntary work or housework, although most authors highlight the importance of such measurements.^7^

Moreover, from researchers working on this issue, there is a generalized need for validated measurement tools for work productivity and techniques to translate these measurements into costs.^8^

The WPAI-GH (Work Productivity and Activity Impairment – General Health) questionnaire measures the effects of health in general and specific symptoms on work productivity and outside of work.^9^ It is the instrument that has been most assessed and most frequently used.^5^ It has already had its validity and reliability evaluated in relation to several diseases, such as allergies,^10^ dermatitis,^11^ gastroesophageal reflux,^12^ nocturia,^13^ asthma^14^ and irritable bowel syndrome.^15^ This questionnaire can detect absenteeism (days or hours of work missed due to a health problem) and presenteeism (reduction of effectiveness of a person while working, due to a health problem).^16^ The productivity loss would thus be the total work impairment; the sum of absenteeism and presenteeism.

## OBJECTIVE

The aim of our study was to translate, culturally adapt and evaluate the performance of the psychometric properties of WPAI-GH among a Brazilian population.

## METHODS

### WPAI-GH productivity assessment instrument

The WPAI-GH is composed of six questions that ask the subject if he is currently employed, the number of nonworking hours due to health problems, the number of nonworking hours due to other reasons (e.g. vacation), the number of hours really worked, how much the health problems affected his productivity while he was working and how much the health problems affected his daily activities over the last seven days. The two last questions are evaluated on a scale of 10 points, ranging from 0 (no effect on work) to 10 (health problems prevent the person from working).^17^

The questions are computed according to specific calculation rules and have four scores: (1) percentage work time missed due to health (absenteeism); (2) percentage impairment at work due to health (presenteeism); (3) percentage overall work productivity loss due to health (absenteeism and presenteeism); and (4) percentage daily activity impairment outside of work due to health. High scores indicate prolonged sick leave or impairment and decreased productivity.^9^The score from this questionnaire can be transformed into monetary value. The general percentage work limitation score is multiplied by the employee's hourly wage to determine the value of the productivity loss.

### Translation and cross-cultural adaptation

The translation and cross-cultural adaptation of the instrument were undertaken on the basis of the directions in da Mota Falcão et al.,^18^ consisting of translation, back-translation and cross-cultural adaptation. The Brazilian Portuguese version of the WPAI-GH questionnaire is presented in the Annex below.

### Initial translation

The items of the WPAI-GH version were initially translated by an independent Brazilian health professional with great knowledge of the English language who was aware of the research objectives. The importance of the conceptual translation was emphasized to the detriment of a strictly literal translation. We thus obtained the first Portuguese version.

### Back-translation and evaluation of the initial translation

The initial translation (first version in Portuguese) was back-translated into English by an English teacher who had not participated in the previous stage. The original instrument was then compared with the new English version. A multi-disciplinary group composed of four health professionals (one rheumatologist, two dentists and a nurse) documented and analyzed the discrepancies that were found. Some verb tenses and sentences in Portuguese were rewritten until a consensus was reached. Thus, the second Portuguese version was obtained.

### Evaluation of the cross-cultural adaptation

The questionnaire was applied to a group of 10 patients randomly selected at the rheumatology outpatient clinic of Universidade Federal de São Paulo — Escola Paulista de Medicina (Unifesp-EPM). To evaluate the degree of comprehension of the questions, we selected the ones that did not appear to be easy to comprehend. The same group as before evaluated these items with the aim of replacing them with others expressing the same notion but easier to comprehend, even using suggestions from the patients themselves, but trying not to change the structure and evaluative properties of these questions. This new version was then applied to a group of 10 patients randomly selected at the same place. Its cultural equivalence was tested again until none of the items presented comprehension problems. Thus, the third Portuguese version was obtained.

### Evaluation of the psychometric properties

After finishing the process of translation and cross-cultural adaptation of the instrument, we assessed its reliability and validity.^19^ One hundred subjects who were currently employed and complaining of a health problem that in their opinion affected their performance at work and/or outside of work were included as participants in the survey. The study was approved by the Research Ethics Committee of Universidade Federal de São Paulo and the information was collected after obtaining written consent from the subjects.

### Reliability: test-retest evaluation

The reliability of the Portuguese version of the WPAI-GH questionnaire was assessed by means of two interviews.

A group of 100 subjects formed by employees of São Paulo Hospital and patients at the outpatient clinic of the Rheumatology division of Unifesp-EPM was selected and assessed by two interviewers. Two assessments were made by one or other of these two observers on the same day. The second assessment by the same observer was made at most one hour after the first assessment.

### Validity

The same group of 100 subjects was interviewed in order to assess the construct validity. The WPAI-GH questionnaire was assessed by correlating it with other sociodemographic and clinical parameters related to their activities, such as length of work, absences from work and degree of damage, among others. These parameters were collected during the first interview with the patient.

In addition to the abovementioned parameters, the instrument was compared with the Short Form-36 (SF-36) generic quality-of-life evaluation questionnaire (Medical Outcomes Study 36-Item Short-Form Health Survey), which evaluates the domains of: general health perceptions, physical functioning, role limitations due to physical health, role limitations due to emotional health, bodily pain, vitality, social functioning and general mental health. The score for each domain ranges from 0 to 100, such that 0 is the worst status and 100 is the best. The SF-36 instrument had already been translated into Brazilian Portuguese and its psychometric properties had already been tested.^20^

### Questionnaire administration

The questionnaires were individually administered to employees of São Paulo Hospital and to patients at the outpatient clinic of the Rheumatology division of Unifesp-EPM.

The employees were approached at their workplace and patients while being attended, by two postgraduate students who explained the objectives and content of the questionnaire and asked for their help and consent to participate in the survey. First of all, the researchers asked if the subject was presently employed and whether he/she had felt any symptom or health problem during the last seven days. If the person gave responses compatible with the inclusion criteria of the study and had time to answer the questionnaires immediately, the researchers then asked the subject to complete a form with sociodemographic data. Following this, they applied the WPAI-GH for the first time in the form of an interview. Next, the SF-36 was administered in the form of an interview as well. The researchers then asked the interviewees to wait for about 25-30 minutes for the last questionnaire to be administered. A randomization table was then used to determine whether the second administration of the WPAI-GH questionnaire would be self-administered or interviewer-administered. When it was self-administered, the researchers waited while the participants answered the questions and the questionnaires were immediately collected after completion. No form of incentive or bonus payment was offered to the participants in the study.

### Statistical analysis

The data were analyzed by means of the Statistical Package for the Social Science (SPSS) program, version 11.0. Descriptive statistics was compiled for sociodemographically and clinically characterizing the study population.

The intraclass correlation coefficient and Cronbach's alpha were used to assess the reliability and internal consistency of the instrument. Pearson's correlation coefficient was used to assess its validity.

The analyses on the subjects of the sample were made firstly as a single group and then as two separate groups according to the method used for administering the second questionnaire (interviewer-administered or self-administered).

## RESULTS

### Cross-cultural adaptation

No question was considered non-applicable. The patients showed good comprehension of the questions with some suggestions and it was not necessary to make any substitution or alteration of any question already assessed in the second Portuguese version.

### Evaluation of the measurement properties

#### Characteristics of the population studied

Table 1 shows the sociodemographic characteristics of the 100 patients included in the reliability and validity assessment of the Portuguese version of the WPAI-GH questionnaire. Eighty-two per cent of the subjects were women, with mean age of 39.22 years (standard deviation, SD: 11.17). With regard to their schooling level, 27% had completed intermediate school; 34%, high school and 39%, college degree.

**Table 1:** Distribution of sociodemographic and clinical characteristics of the study sample

Sociodemographic and clinical characteristics	(n = 100)	
**Gender**		
Male	18	18%
Female	82	82%
**Age** (years)		
Mean (SD)	39.22	(± 11.17)
Median (minimum-maximum)	40	(18-64)
**Education** (n; %)		
Intermediate school	27	27%
High school	34	34%
College degree	39	39%
**Years of education**		
Mean (SD)	11.87	(± 5.51)
Median (minimum-maximum)	11	(0.5-25)
**Length of employment** (years)		
Mean (SD)	8.42	(± 8.09)
Median (minimum-maximum)	5	(0.08-30)
**Hours of work per week**		
Mean (SD)	41.74	(± 10.54)
Median (minimum-maximum)	40	(16-84)
**Monthly wage** (reais)		
Mean (SD)	1145.81	(± 993.90)
Median (minimum-maximum)	825	(200-5000)
**Absenteeism** (n; %)		
Yes	16	16%
No	84	84%
**Missing days from work** (n = 16)		
Mean (SD)	1.44	(± 0.63)
Median (minimum-maximum)	1	(1-3)
**Health interference with job functioning** (n; %)		
Yes	77	77%
No	23	23%
**Intensity of the interference** (n; %)		
A great deal of the time	6	7.80%
A lot of the time	19	24.70%
From time to time	5	6.50%
Only occasionally	47	61%
**Medical conditions** (n; %)		
Gastrointestinal diseases	4	4%
Infectious diseases	13	13%
Musculoskeletal diseases	49	49%
Neuropsychological diseases	28	28%
Oncological diseases	2	2%
Respiratory diseases	4	4%
**Length of symptoms** (years)		
Mean (SD)	1.76	(± 4.07)
Median (minimum-maximum)	0.08	(0.002-25)
**Current medication** (n; %)		
With prescription	40	40%
Without prescription	24	24%
With/without prescription	4	4%
No medication	32	32%

*SD = standard deviation*

Table 1 shows some clinical characteristics of the sample and their impact on the productivity of these patients at work. Even though only 16% of the subjects had missed work during the last week, and had had on average 1.44 days of absence, 77% of them said that their health problems interfered with their work activities. It is important to say that, in this population, the intensity of health interference at work was mild; of the 100 patients assessed, 61% classified this interference as not much. Most patients showed musculoskeletal diseases (49%) and the medication in use had been prescribed in 40% of the cases.

Table 2 shows the values obtained for each domain of the WPAI-GH version, in the Portuguese language. Most mean values for questionnaire domains were between 40 and 50, except for the first domain, which measures absenteeism. The highest mean values were the ones obtained for the domain of activity impairment due to health (49.70). This represents almost 50% impairment of the individual's capacity to perform regular activities outside of work.

**Table 2:** Work Productivity and Activity Impairment — General Health (WPAI-GH) questionnaire scaling test results

Scale	Mean	Standard deviation	Minimum	Maximum
Work time missed (%)	8.40	15.33	0.00	100.00
Impairment at work (%)	43.30	27.01	0.00	100.00
Overall work productivity loss (%)	47.46	27.34	0.00	100.00
Activity impairment (%)	49.70	31.28	0.00	100.00

Table 3 shows the values obtained for each domain of SF-36. Most mean values for the domains of this questionnaire were between 50 and 70, except for the physical functioning and bodily pain domains, which showed lower scores.

**Table 3:** Short-form-36 (SF-36) questionnaire scaling test results

Scale	Mean	Standard deviation	Minimum	Maximum
Physical functioning	68.15	27.14	5.00	100.00
Role limitations due to physical health	42.00	39.87	0.00	100.00
Bodily pain	48.92	25.07	0.00	100.00
General health perceptions	69.39	24.00	10.00	100.00
Vitality	55.40	22.07	0.00	95.00
Social functioning	66.38	25.23	0.00	100.00
Role limitations due to emotional health	59.33	40.37	0.00	100.00
General mental health	68.08	20.81	4.00	100.00

#### Reliability

The overall internal consistency, as assessed by Cronbach's alpha, was 0.74. Table 4 shows the reliability results obtained by means of test-retest evaluation for each domain of the Portuguese version of the WPAI-GH questionnaire, using the intraclass correlation coefficient and Cronbach's alpha.

**Table 4:** Intraclass and Cronbach's alpha correlation coefficients between first and second administrations of the Work Productivity and Activity Impairment - General Health (WPAI-GH) questionnaire

Scale	Correlation coefficients	
	Intraclass	Cronbach's alpha
Work time missed (%)	0.78*	0.80†
Impairment at work (%)	0.89†	0.89†
Overall work productivity loss (%)	0.90†	0.90†
Activity impairment (%)	0.81†	0.81†

* *Good correlation (0.6 – 0.8);*

† *Excellent correlation (0.8 - 1). There were no findings of either satisfactory correlation (0.4 – 0.6) or weak correlation (0.2 – 0.4).*

The reliability of the four domains of the WPAI-GH was considered highly satisfactory. All domains showed an excellent correlation between the first and second administrations of the questionnaire, independent of the administration method used on the second occasion, except for the domain of work time missed due to health, which indicated a good intraclass correlation.

Table 5 shows the results from the reliability assessment when the second method of applying the questionnaire was modified. These reliability results were also obtained by means questionnaire scaling test results of test-retest evaluation for each domain of the Portuguese version of the WPAI-GH questionnaire, using the intraclass correlation coefficient and Cronbach's alpha.

**Table 5:** Intraclass and Cronbach's alpha correlation coefficients between first and second administrations of the Work Productivity and Activity Impairment - General Health (WPAI-GH) questionnaire by administration method

Scale	Interview/self-administered		Interview/interview	
	Intraclass	Cronbach's alpha	Intraclass	Cronbach's alpha
Work time missed (%)	0.76*	0.79*	0.83†	0.84†
Impairment at work (%)	0.91†	0.91†	0.88†	0.88†
Overall work productivity loss (%)	0.92†	0.93†	0.88†	0.88†
Activity impairment (%)	0.77*	0.78*	0.85†	0.85†

* *Good correlation (0.6 – 0.8);*

† *Excellent correlation (0.8 - 1). There were no findings of either satisfactory correlation (0.4 – 0.6) or weak correlation (0.2 – 0.4).*

For the administration sequence of interview followed by self-administration, the reliability was shown to be very satisfactory. The domains of impairment at work due to health and overall work productivity loss due to health showed excellent correlation. The domains of work time missed due to health and of activity impairment due to health indicated good correlation.

For the administration sequence of interview followed by interview, the reliability was shown to be highly satisfactory. All domains showed an excellent correlation. As expected, the administration method of interview followed by interview produced better reliability, because the administration method was not modified.

#### Validity

Table 6 shows Pearson's correlation coefficients for the sociodemographic data and the four domains of the Portuguese version of the WPAI-GH questionnaire.

**Table 6:** Pearson's correlation coefficient between sociodemographic and clinical characteristics in relation to Work Productivity and Activity Impairment - General Health (WPAI-GH) questionnaire scales

		WPAI-GH scales		
Sociodemographic and clinical characteristics	Work time missed (%)	Impairment at work (%)	Overall work productivity loss (%)	Activity impairment (%)
**Gender**				
Pearson's correlation	-0.12	0.096	0.009	0.322	
p-value	0.233	0.341	0.928	0.001*
**Age**				
Pearson's correlation	0.032	0.15	0.127	0.116
p-value	0.753	0.137	0.208	0.251
**Education**				
Pearson's correlation	0.004	-0.163	-0.136	0.269
p-value	0.968	0.104	0.177	0.007†
**Years of education**				
Pearson's correlation	0.035	-0.198	-0.151	-0.299
p-value	0.732	0.049‡	0.134	0.003†
**Length of employment**				
Pearson's correlation	0.128	0.099	0.133	0.039
p-value	0.203	0.328	0.187	0.699
**Hours of work per week**				
Pearson's correlation	0.013	0.317	0.261	0.291
p-value	0.901	0.001*	0.009†	0.003†
**Monthly wage**				
Pearson's correlation	0.200	-0.057	0.046	0.133
p-value	0.046‡	0.576	0.65	0.188
**Absenteeism**				
Pearson's correlation	0.511	0.19	0.323	0.066
p-value	0.00*	0.058	0.001*	0.517
**Missing days from work**				
Pearson's correlation	0.405	0.134	0.238	0.080
p-value	0.000*	0.183	0.017‡	0.429
**Health interference with job functioning**				
Pearson's correlation	0.137	0.315	0.325	0.125
p-value	0.173	0.001*	0.001*	0.217
**Intensity of the interference**				
Pearson's correlation	-0.169	-0.544	-0.509	-0.455
p-value	0.142	0.000*	0.00*	0.000*
**Symptom duration**				
Pearson's correlation	0.066	0.065	0.086	0.189
p-value	0.513	0.518	0.393	0.059
**Current medication**				
Pearson's correlation	-0.130	-0.067	-0.118	-0.023
p-value	0.196	0.505	0.242	0.821

* *= p < 0.001;*

† *= p < 0.01;*

‡ *= p < 0.05.*

Statistically significant correlation coefficients were found between the following variables: sex, schooling, years of study and weekly schedule, and the following domains: impairment at work, overall work productivity loss and activity impairment. There was also a statistically significant correlation between the domain of work time missed due to health and the variable of monthly salary.

Table 6 also shows Pearson's correlation coefficients for the clinical data and the four domains of the Portuguese version of the WPAI-GH questionnaire.

Statistically significant correlation coefficients were found between the following variables: absence from work, days of absence, health interference at work and intensity of the interference, and all four domains of WPAIGH. There was also a statistically significant correlation between the domain of work time missed due to health and the variable of absence from work, and between the domains of impairment at work and overall work productivity loss and the variable of intensity of the interference.

Table 7 shows the construct validity analysis, the correlations between the four domains of WPAI-GH and the eight domains of SF-36.

**Table 7:** Construct validity analysis: Pearson's correlation coefficient between SF-36 questionnaire scales and Work Productivity and Activity Impairment-General Health (WPAI-GH) questionnaire scales

			WPAI-GH scales	
SF-36 Scales	Work time missed (%)	Impairment at work (%)	Overall work productivity loss (%)	Activity impairment (%)
**Physical functioning**				
Pearson's correlation	-0.079	-0.371	-0.352	-0.367
p-value	0.435	0.000*	0.000*	0.000*
**Role limitations due to physical health**				
Pearson's correlation	-0.035	-0.358	-0.315	-0.435
p-value	0.727	0.000*	0.000*	0.000*
**Bodily pain**				
Pearson's correlation	-0.055	-0.472	-0.445	-0.420
p-value	0.587	0.000*	0.000*	0.000*
**General health perceptions**				
Pearson's correlation	-0.052	-0.224	-0.200	-0.198
p-value	0.608	0.025†	0.046†	0.049†
**Vitality**				
Pearson's correlation	-0.067	-0.310	-0.264	-0.327
p-value	0.510	0.002‡	0.008‡	0.000*
**Social functioning**				
Pearson's correlation	-0.095	-0.406	-0.377	-0.243
p-value	0.348	0.000*	0.000*	0.015†
**Role limitations due to emotional health**				
Pearson's correlation	-0.109	-0.206	-0.181	-0.170
p-value	0.278	0.040†	0.072	0.091
**General mental health**				
Pearson's correlation	-0.174	-0.343	-0.317	-0.172
p-value	0.084	0.000*	0.000*	0.086

* *= p < 0.001;*

† *= p < 0.05;*

‡ *= p < 0.01.*

Statistically significant correlation coefficients were found between the following domains: impairment at work, overall work The WPAI-GH scores range from lower to comparisons between the questionnaires show productivity loss and activity impairment, higher values according to the impairment or negative correlation coefficients. and all the domains of SF-36.

The WPAI-GH scores range from lower to higher values according to the impairment or worsening of productivity. On the contrary, the SF-36 scores range from lower to higher value according to the improvement in the patient's general health status. This is the reason why the comparisons between the questionnaires show negative correlation coefficients.

## DISCUSSION

It is still difficult to measure work pro-pain in SF-36. general health status. This is the reason why the ductivity losses caused by health problems. Despite the importance given to this issue over the last few years, and despite the existence of several generic and specific instruments for performing this task, most instruments are available only in the English language.

Translation, cross-cultural adaptation and validation of these instruments is a hard task because of its pioneering nature and because there are no similar instruments in Brazil with which to make the necessary comparisons for this process.

Since the WPAI-GH questionnaire has simple and objective questions, the interviewees did not have any difficulty in understanding the questions. Another important factor is that in some questions, such as numbers 5 and 6, the instrument itself gives examples of some situations, which facilitated comprehension among the subjects who filled in the questionnaire.

In the reliability assessment, we observed highly satisfactory agreement scores with regard to the administration methods and internal consistency of the instrument, for the whole sample and also when it was subdivided according to the administration method on the second occasion. This shows that both administration methods (interview or self-administration) are reliable. In another study involving reliability assessments on instruments among a population with low schooling levels, it was observed that it was only better to apply the instrument in the self-administered form when the subjects had completed intermediate school, because the level of confidence was higher in this situation.^21^

In the present study, we cannot prove whether the subjects with lower schooling levels would have been able to answer the questionnaire in the self-administered form, since we had a higher number of subjects with schooling levels up to eighth grade selected for interview. Although they showed that there was an excellent correlation between the administration methods, we cannot conclude that they would have had the same performance if the administration had been via the self-administered method.

In order to evaluate the construct validity of the WPAI-GH questionnaire we needed to compare it with a generic instrument for quality of life. We believed that the SF-36 was the best option for this.

We observed that the domains most related to the physical component, such as physical functioning, role limitations due to physical health, bodily pain and general health status, showed the best correlations with the WPAI-GH domains; except for the domain of work time missed due to health, which did not correlate with any domain. We attribute this finding to the fact that the number of nonworking days was very small in the studied sample (1.44 on average), thus giving a lower score to the domain of work time missed due to health, which showed a mean score of 8.40 on a scale from 0 to 100; very different from the other three WPAI-GH domains, which showed scores of more than 40.

Many patients said that, even though they were having some health problems, they did not stop going to work and tried to accomplish their tasks in the best possible way. They thought that the highest damage occurred during tasks outside of the workplace. This shows the need for instruments that measure presenteeism, because simply measuring the number of days absent from work would not be enough to check the impact of a health problem on the subject's productivity. Absenteeism represents only a small proportion of the total indirect costs.^3^

Evaluation of productivity losses makes it possible for an assessment of disease costs to incorporate a better approach towards the indirect costs, which in some situations exceed the direct cost. It would be of great value to carry out local studies that use specific instruments to evaluate the impact of some diseases on productivity losses among workers, because other instruments measuring productivity losses would thus be made available for comparison purposes.

## CONCLUSIONS

The Brazilian Portuguese version of the WPAI-GH questionnaire is a reliable and valid measurement tool and may be useful for those who seek to measure the impact on productivity of health problems among populations of Brazilian employees.
