# Erratum

**DOI:** 10.1590/S1516-31802007000300015

**Published:** 2007-05-03

In the paper “The Brazilian Portuguese version of the Work Productivity and Activity Impairment – General Health (WPAI-GH) Questionnaire” by Ciconelli RM, Soárez PC, Kowalski CC, Ferraz MB [Sao Paulo Med J. 2006;124(6):325-32].

In question (6), the sentence “Problemas de saúde não afetaram meu trabalho” should be changed to “Problemas de saúde não afetaram minhas atividades regulares”, and the sentence “Problemas de saúde me impediram de fazer meu trabalho” should be changed to “Problemas de saúde me impediram de fazer minhas atividades regulares”.

The revised sentences in their context are as follows:

**Table:** 

Problemas de saúde não afetaram minhas atividades regulares	$\bar{\begin{array}{c} \;0\;1\;2\;3\;4\;5\;6\;7\;8\;9\;10\;\\ \text{CIRCULE UM NÚMERO} \end{array}}$	Problemas de saúde me impediram de fazer minhas atividades regulares

